# Free groin flap for recurrent severe contractures of the neck in children

**DOI:** 10.4103/0970-0358.70722

**Published:** 2010-09

**Authors:** Abhishek Ghosh, R. Jayakumar

**Affiliations:** Department of Plastic Surgery, Specialist Hospital, Kochi, India

**Keywords:** Free flap, recurrent post burns contracture neck

## Abstract

**Context:**

Severe post burns contracture in children not only leads to functional impairment but also has profound psychological impact on the child. Untreated neck contractures have been shown to inhibit mandibular growth. Skin grafting in children has a higher rate of recurrence and in these cases a thin pliable flap seems to provide a durable solution.

**Aim:**

To study the feasibility of using primarily thinned free groin flap in the treatment of recurrent neck contractures in children.

**Materials and Methods:**

Five patients, in the age group of 5–10 years, with recurrent neck contractures and operated between 2005 and 2008 were included in this study. The sternomental distance, lateral flexion angle and cervicomental angle were measured preoperatively, postoperatively and during the subsequent follow-up visits. The patients were followed up for a period between 1 and 3 years with a mean of 29 months.

**Results:**

All the flaps survived. The cervicomental angle improved significantly to 90–105°, the lateral flexion angle improved to 35–45° and the sternomental distance increased considerably.

**Conclusions:**

Recurrent post burns contracture of the neck in children causes not only functional and aesthetic impairment but also psychological problems. A free microthinned groin flap provides a very attractive solution for this problem and should be seen as an effective alternative in recurrent cases.

## INTRODUCTION

Severe post burns contracture of neck remains a daunting challenge for any reconstructive surgeon. It produces functional restriction of movements, hampering the daily activities. It produces severe disfigurement and the resultant psychosocial issues. The airway access is hampered and the neck contracture needs to be addressed on a priority basis for subsequent reconstructions. If untreated, neck contractures in children can lead to inhibition of normal mandibular growth[1–3] and hence it is of utmost importance that a stable and lasting solution be given to these patients. Different techniques for treating scar contractures include skin grafting, local and free flaps and tissue expansion. Skin grafting with splinting is the most common modality of treatment but prolonged splinting is difficult to maintain in adults and more so in children. So, in children it is not uncommon to come across recurrent neck contractures in spite of adequate release and grafting.[4–6] It is for these recurrent contractures of the neck that we propose to use the free groin flap.

The free groin flap is one of the earliest free flaps described. A large area of skin can be harvested with minimal donor site morbidity and a two team approach is possible. It had become unpopular due to the anatomical variation of the pedicle, the short length and narrow diameter of the pedicle, and the thickness of the conventionally raised groin flap. We still feel that the groin flap is reliable, easy to harvest and meets all the requirements for reconstructing the neck in post burns contracture of neck. The shortcomings of the groin flap have been overcome by elevating the flap from lateral to medial as a perforator based flap in contrast to the conventional technique of elevating the flap from medial to lateral. This series studies the feasibility of using the free thinned groin flap in treating post burns contractures of the neck in children.

## MATERIALS AND METHODS

**Figure 1:**
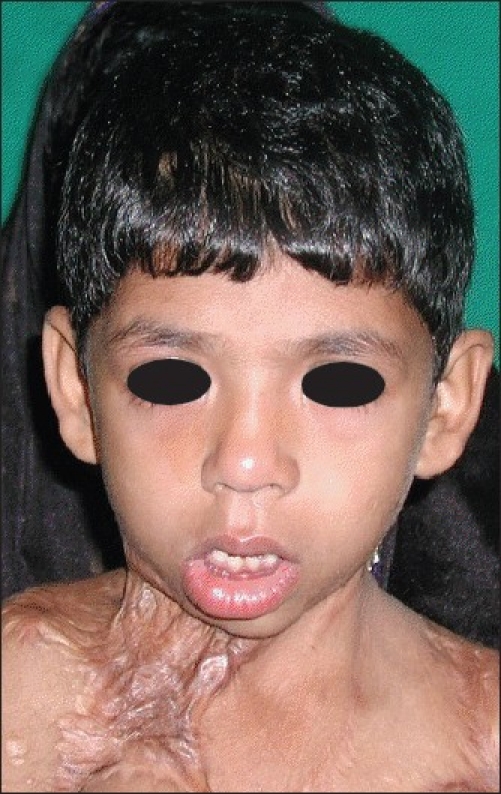
Preoperative photograph of Case 1. A 5 year old boy who had been previously grafted for PBC neck twice in a burns centre with recurrenc

**Figure 2:**
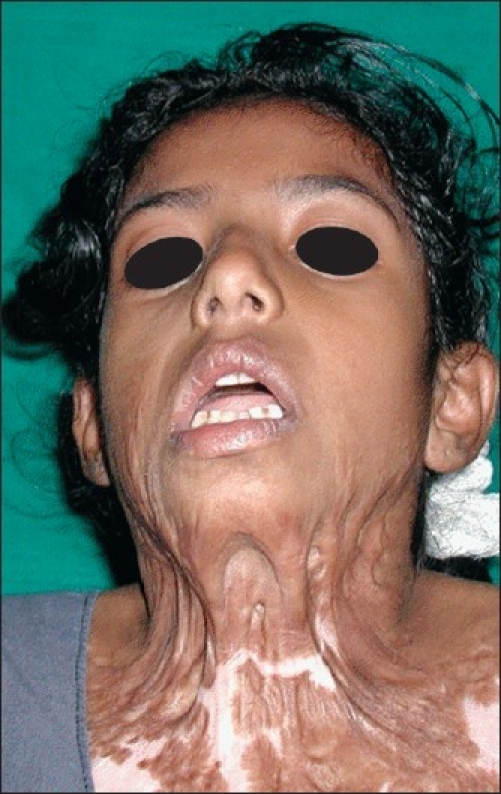
Preoperative photograph of Case 3. An 8 year old girl who had been previously grafted twice in a burns centre with recurrent contracture

We operated on five patients, of age between 5 and 10 years, with severe recurrent neck contractures, during 2005–2008 [Figures 1 and 2]. All of them were operated with skin grafting at least twice in other burn centres but had recurrence. The sternomental distance, lateral flexion angle and cervicomental angle were measured preoperatively, postoperatively and during the subsequent follow-up visits. The patients were followed up for a period between 1 and 3 years with a mean of 29 months [Table 1].

**Table 1:** Preoperative observations

**	*Age (years)*	*Grade*	*Cervicomental angle*	*Lateral flexion*	*Sternomental distance (cm)*
Case 1	5	IV	30°	15°	3
Case 2	7	IV	20°	10°	3
Case 3	8	III	45°	20°	4.5
Case 4	10	IV	30°	15°	4
Case 5	7	III	30°	15°	3.5

### Operative technique

**Figure 3:**
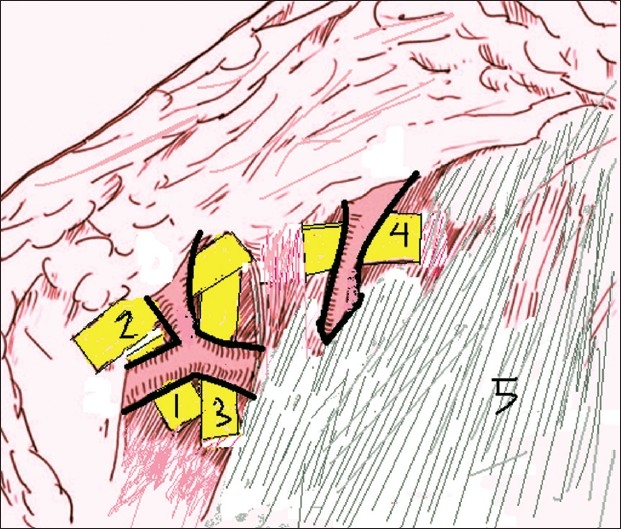
Schematic representation of the elevation of the free groin flap. The perforator to the skin flap has been identified. 1. Main branch, 2. Superficial branch, 3. Deep branch, 4. Perforator from deep branch supplying the skin, 5. Sartorius fascia

**Figure 4:**
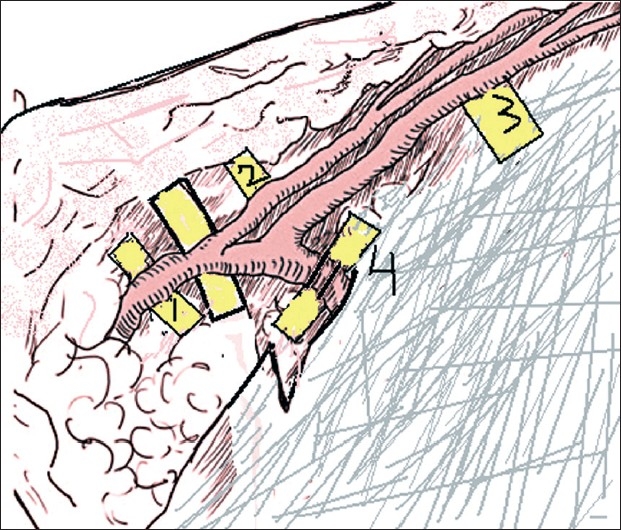
Schematic representation of the elevation of the free groin flap. The perforator to the skin flap has been identified. 1. Main branch, 2. Superficial branch, 3. Deep branch, 4. Perforator from deep branch supplying the skin, 5. Sartorius fascia

The flap is marked as per the lint pattern with the vessel in the centre. In contrast to the conventional technique where the flap is elevated from medial to lateral, we start elevating the flap from lateral to medial. As the flap is raised from lateral side, the perforator to the skin is identified [Figure 3]. This perforator is then skeletonised to its origin. Thus, it does not make any difference whether the perforator is from the superficial or the deep vascular system and the problem of the variability of the pedicle is solved. The perforator thus isolated is dissected distally in a plane between the superficial and deep layers of fat. This dissection is a little tedious and is done under an operating microscope [Figure 4]. With this technique, we obtain a pedicle length of 5–7 cm which is sufficient in most cases. The flap is thinned primarily at the level between the deep and superficial layers of the fat; hence, a thin pliable skin flap is obtained.

**Figure 5:**
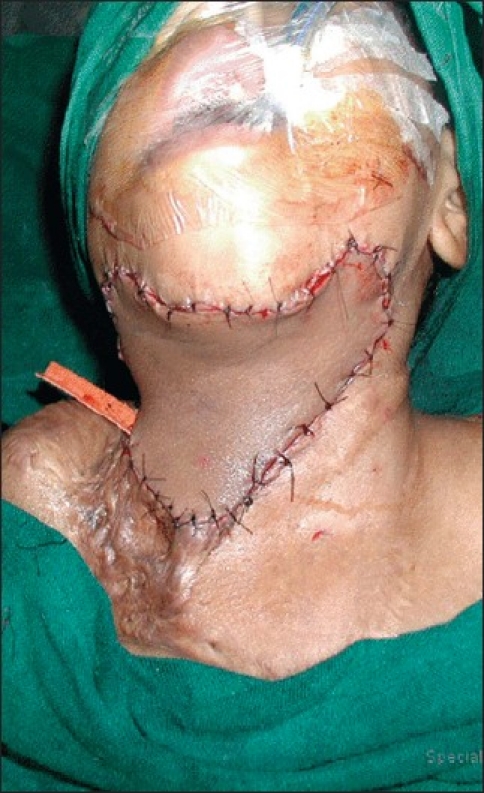
Intra-operative picture of the free groin flap after inset

In all the patients, the contracture with the platysma was entirely released. The free groin flap harvested was used to resurface the defect. The superior thyroid artery and internal jugular vein were used as the recipient pedicle. Flap inset was given with the cervicomental angle in the range of 90–105° and primary neck contours were recreated [Figure 5].

## RESULTS

All the flaps survived. One flap had to be reopened because of venous congestion but was salvaged. There was no partial or total flap loss. The mean pedicle length was 5.5 cm and the flap dimensions ranged from 10 × 12 cm to 14 × 20 cm. The mean stay in hospital was for 15 days. The cervicomental angle improved significantly to 90–105°, the lateral flexion angle improved to 35–45° and the sternomental distance increased considerably [Table 2].

**Figure 6:**
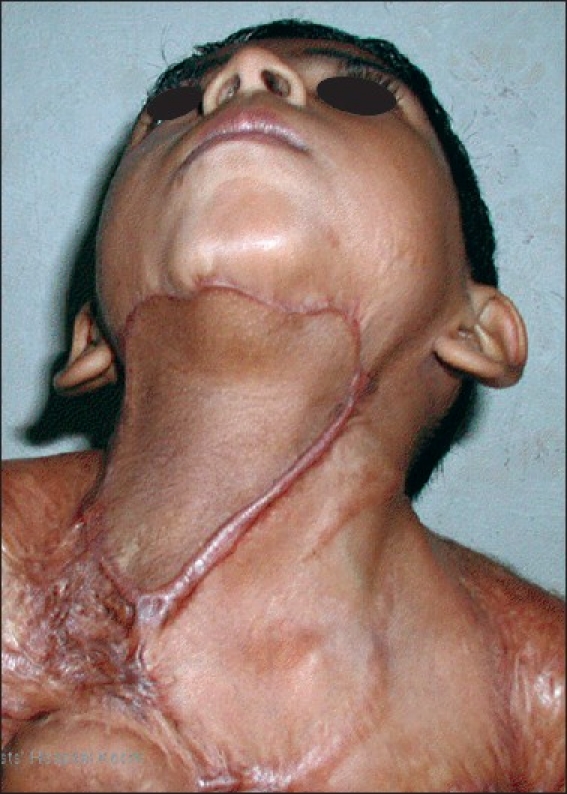
Postoperative photograph at 18 months of follow-up. There is no recurrence of the contracture nad the cervicomental angle is maintained

**Figure 7:**
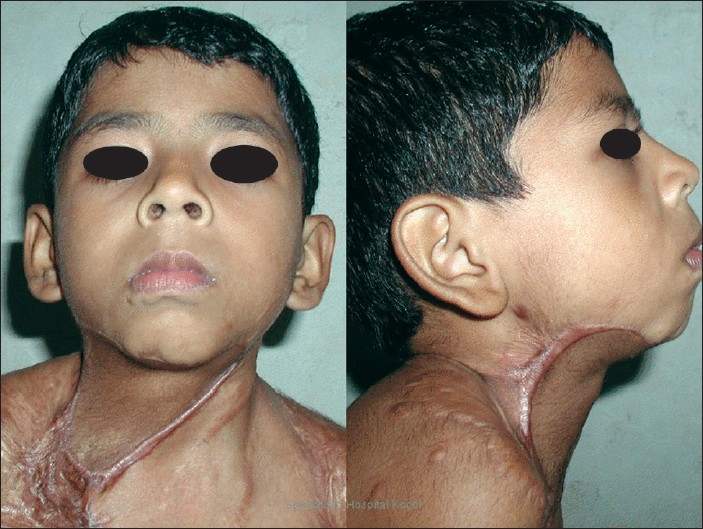
Postoperative picture at 24 months of follow-up with no recurrence

**Figure 8:**
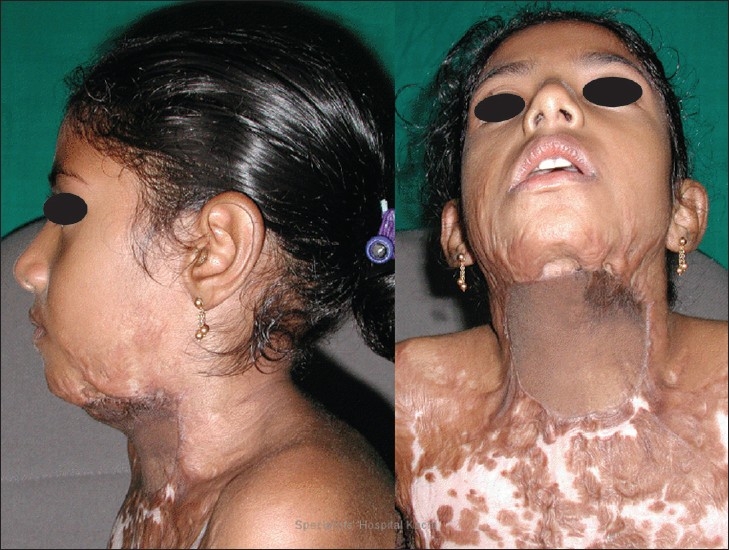
Postoperative picture at 12 months of follow-up with no recurrence

**Figure 9:**
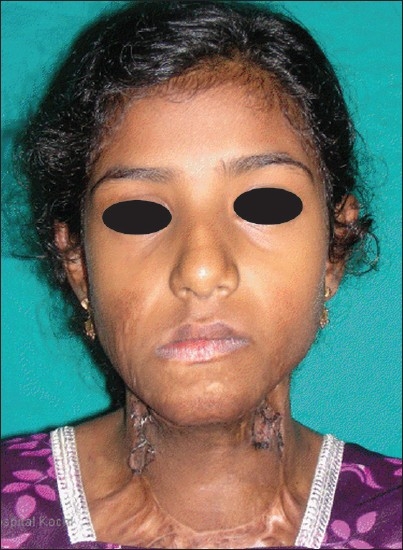
Postoperative picture at 36 months of follow-up with maintained neck contours and no recurrence

All the observations were maintained in subsequent follow-ups and there was no recurrence [Figures 6–9].

**Table 2:** Postoperative observations

**	*Age (years)*	*Grade*	*Cervicomental angle*	*Lateral flexion*	*Sternomental distance (cm)*
Case 1	5	IV	100°	40°	7
Case 2	7	IV	90°	40°	7.5
Case 3	8	III	105°	45°	7.5
Case 4	10	IV	100°	40°	8
Case 5	7	III	100°	40°	6.5

## DISCUSSION

The primary objectives in reconstructing severe neck contracture are

- to release the contracture completely with restoration of range of movements,
- to restore the contour of the cervico-mental angle,
- to maintain the aesthetic subunits of the neck and
- to prevent re-contracture.

In children, it is particularly important to release neck contractures early as they may inhibit mandibular development.[1–3] Daily activities become a problem and there is huge psychological stress due to this deformity not only for the child but also for the parents.[7–9] Hence, it is imperative to provide a solution which satisfies all the above criteria perfectly.

Skin grafts need prolonged splinting which is very difficult to maintain in a child and hence are prone for recurrence. Local flaps with tissue expansion require multiple surgeries and is time consuming and expensive. It is not well tolerated and creates a temporary disfigurement which is difficult for children to comprehend and accept. Tissue expanders in the neck also carry a high complication rate with implant exposure and buckling being the most common.[10] In this scenario, a free flap which gives an excellent functional and aesthetic outcome in a single surgery without the need for postoperative splinting seems ideal for reconstruction in children.

In our opinion, a free groin flap meets all the desired parameters for management of severe contractures of neck. It is possible to take a large flap from the groin area even in children where other flaps may not be suitable. Also, the groin region is rarely affected in a burnt patient and a flap can be harvested with ease. We have solved the problem of variability of the pedicle and the bulk of a conventional groin flap by raising the flap from lateral to medial and identifying the perforator to the skin first. The skin over the groin is thin and the flap can be raised in a subcutaneous plane, providing a thin and pliable skin flap. The raising of the free groin flap in the subcutaneous plane by following the technique described above provides excellent recreation of neck contours and cervico-mental angle. The donor area can be closed primarily or may need small graft which is well hidden. There is no need for prolonged and cumbersome splinting and there is no recurrence.

## CONCLUSION

Recurrent post burns contracture of the neck is a very difficult condition to treat. Contractures in children need to be addressed on a priority basis as they cause not only functional and aesthetic impairment but also psychological problems. A free micro-thinned groin flap provides a very attractive solution for this problem and should be seen as an effective alternative in recurrent cases.
